# Role of Natural Killer Cells during Pregnancy and Related Complications

**DOI:** 10.3390/biom12010068

**Published:** 2022-01-04

**Authors:** Deviyani Mahajan, Neeta Raj Sharma, Sudhakar Kancharla, Prachetha Kolli, Ashutosh Tripathy, Amarish Kumar Sharma, Sanjeev Singh, Sudarshan Kumar, Ashok Kumar Mohanty, Manoj Kumar Jena

**Affiliations:** 1 Department of Biotechnology, School of Bioengineering and Biosciences, Lovely Professional University, Phagwara 144411, Punjab, India; deviyani.11919673@lpu.in (D.M.); neeta.raj@lpu.co.in (N.R.S.); ashutosh.11701104@lpu.in (A.T.); amarish.19824@lpu.co.in (A.K.S.); sanjeev.15935@lpu.co.in (S.S.); 2 Devansh Lab Werks, 234 Aquarius Drive, Homewood, AL 35209, USA; sudhakar@devlabwerks.com; 3 Microgen Health Inc., 14225 Sullyfield Cir Suite E, Chantilly, VA 20151, USA; Prachetha@microgenhealth.com; 4 Animal Biotechnology Centre, National Dairy Research Institute, Karnal 132001, Haryana, India; kumarsudershan@gmail.com; 5 Indian Veterinary Research Institute, Mukteswar Campus, Mukteswar 263138, Uttarakhand, India; ashokmohanty1@gmail.com

**Keywords:** naturalkiller cells, endometrium, pregnancy, decidual angiogenesis, preeclampsia

## Abstract

A high number of leucocytes reside in the human endometrium and are distributed differentially during the menstrual cycle and pregnancy. During early pregnancy, decidual natural killer (dNK) cells are the most common type of natural killer (NK) cells in the uterus. The increase in the number of uterine NK (uNK) cells during the mid-secretory phase of the menstrual cycle, followed by further increase of dNK cells in early pregnancy, has heightened interest in their involvement during pregnancy. Extensive research has revealed various roles of dNK cells during pregnancy including the formation of new blood vessels, migration of trophoblasts, and immunological tolerance. The present review article is focused on the significance of NK cells during pregnancy and their role in pregnancy-related diseases. The article will provide an in-depth review of cellular and molecular interactions during pregnancy and related disorders, with NK cells playing a pivotal role. Moreover, this study will help researchers to understand the physiology of normal pregnancy and related complications with respect to NK cells, so that future research work can be designed to alleviate the complications.

## 1. Introduction

For decades, immunologists have been captivated by the link between mother and fetus. Billingham and colleagues for the first time described the survival of the semi-allogeneic fetus in 1953 as an illustration of mother’s immune tolerance to the fetus [1]. Since then, various theories have been put forward to explain this process, including the original theories of the fetus antigenic immaturity, inertness of the maternal immune system, and the presence of an anatomical barrier between the mother and the embryo. All of these theories have been proven false and in the recent years, the concept of active immune crosstalk has gained popularity, which supports the idea that the immune system at the implantation site is not inhibited; rather, it is active, functional, and carefully managed [2].Recent research findings suggest that the fetal cells and the mother’s immune cells including innate lymphoid cells (ILCs), T regulatory cells, macrophages, etc., are engaged in a bidirectional immune conversation [3,4].

The ILCs are a diverse group of cells that live in all the tissues. Unlike T and B lymphocytes, ILCs do not express diverse antigen receptors (i.e., recombination activation genes-dependent somatic rearrangement) [5]. They have the ability to respond quickly to viral-infected and altered cells. ILCs were recently categorized into five subgroups based upon the transcription factors and cytokine production: ILC1, ILC2, ILC3, lymphoid tissue-inducer (LTi) cells, and the conventional natural killer cells. The ILC1 cells are T-bet^+^ (an immune cell transcriptional factor), and secrete type1 cytokines including interferon-gamma (IFN-γ), tumor necrosis factor-alpha (TNF-α), and GM-CSF. The ILC-2 cells are GATA3^+^, and secrete type2 cytokines interleukin-5 and -13, whereas ILC-3 cells are RORγT^+^ (CCR6 chemokine receptor’s transcription factor required for the development and expression) secreting interleukin-17. Similar to the ILC-3, LTi cells are also RORγT^+^ and play a role in secondary lymphoid formation. The conventional NK cells, such as ILC-1, produce IFN-γ but they have far higher cytotoxicity effects, distinguishing them from the ILC-1 [6].

In non-lymphoid tissues including skin, lungs, and uterus, two subgroups of the natural killer (NK) cells, namely, tissue resistance natural killer (trNK) cells and circulating natural killer (cNK) cells, have been identified in a murine model [7]. In additional, the uterus of the virgin mouse has a considerable number of trNK cells and a small number of cNK cells which are generally considered as insignificant. During pregnancy, however, due to cyclic exposure to sex steroids and invading extravillous trophoblasts, the highly specific uterus tissue comprises ILC1s, cNK cells, and trNK cells which are commonly referred to as uterine NK (uNK) cells to encompass all subsets [5,8].

In humans, NK cells are a highly specialized subset of large granular lymphocytes that roughly make up about 15% of all circulating lymphocytes [9]. They are the large cytotoxic innate lymphoid cells that regulate leukocyte activation and microbial infection surveillance immunologically [10,11]. Because of their potential to distinguish virally-infected and cancerous cells from healthy cells, without being pre-sensitized, they are known as “natural” killers. IFN-γ and TNF-αare two inflammatory mediators secreted by NK cells, which mediate cytotoxicity and produce enormous amounts of inflammatory cytokines [12]. Extensive research has described the role of decidual NK (dNK) cellsin the formation of new blood vessels, trophoblast migration, and immune tolerance in the decidua during pregnancy. Hence, this review will provide in-depth information on the role of NK cells during pregnancy and related disorders.

## 2. Development and Origin of NK Cells

### 2.1. Cellular Stages of NK Cell Development

**Figure 1:**
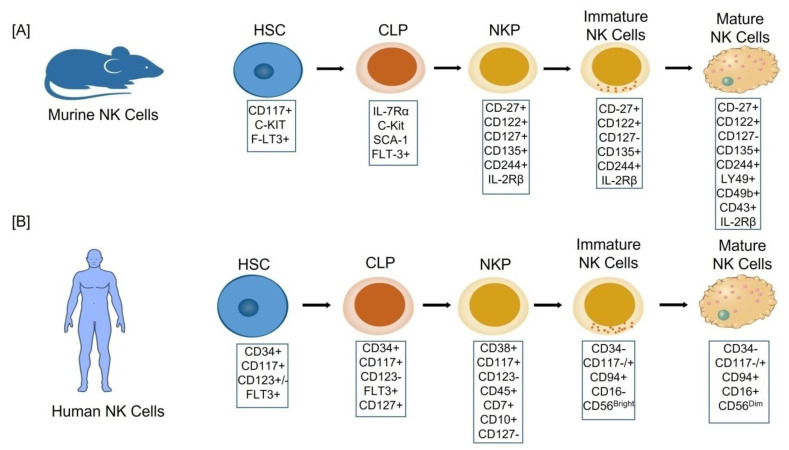
Cellular stages of Natural Killer (NK) cell development: (**A**) murine; (**B**) humans. In both cases the NK cells develop in the bone marrow from the common progenitor hematopoietic stem cells (HSC) and pass through the five different cellular stages to become a mature NK (mNK) cell, each of which is characterized by the different surface markers. CLP: Common lymphoid progenitor cells. NKP: Natural killer progenitor cells.

NK cells are found in the bone marrow, liver, uterus, spleen, and lung, as well asto a lesser extent in secondary lymphoid tissues (SLT), mucosal associated lymphoid tissues (MALT), and the thymus [13]. NK cells, like other blood cells, originate from the bone marrow-derived multipotent progenitor, the CD34^+^ hematopoietic stem cells (HSCs), through a multistage differentiation process defined by the presence of various cell surface markers/receptors [14]. Based upon the presence of activator/inhibitor receptors, adhesion molecules and progressive functional maturation in humans and mice, a linear model of NK cell development has been proposed [15]. Lin-CD27^+^ CD122^+^ CD127^+^ CD135^+^ CD244^+^ has been used to identify a murine bone marrow NK lineage progenitor (NKP) (Figure 1A). NKP stage cells grow into immature NK (iNK) cells after losing CD127 expression and gaining NK1.1 expression [16]. Mature NK cells (mNK) display CD11b, Ly49, CD49b (DX5), and CD43, and are functional with respect to cytotoxicity and IFN-γ production before egressing from the bone marrow [17]. Peripheral NK cells of the mature mouse are further sub-divided into two distinct maturation stages based upon the expression of CD27^+^ and CD27^−^ receptors; the former is a more cytotoxic and cytokine producing group than the latter [18].

Lin-CD34^+^ CD38^+^ CD117^+^ CD123^−^ CD45RA^+^ CD7^+^ CD10^+^ CD127^−^ phenotype, on the other hand, has recently been found in adult human bone marrow tonsils as a restricted NK cell progenitor [19] (Figure 1B). There are five stages in the development of human NK cells:

1. Stage 1: CD34^+^CD117^+^CD123^+/−^FLT3^+^,
2. Stage 2: CD34^+^CD117^+^CD123^−^FLT3^+^CD127^+^,
3. Stage 3: CD38^+^CD117^+^CD123^−^ CD45^+^CD7^+^CD10^+^CD127^−^,
4. Stage 4: (CD34^−^CD117^−/+^CD94^+^CD16^−^) also named as CD56^bright^,
5. Stage 5: (CD34^−^CD117^−/+^CD94^+^CD16^+^) also named as CD56^dim^ [14]

During the transition from CD56^high^ to CD56^low^, the CD56^dim^cells experience a progressive loss of expression of CD94/natural killer group 2 member A (an inhibitory member of the NKG family that is expressed on CD56^high^ NK cells) and Killer Ig-like receptors (KIRs), CD57, and NKG2C (an activating NK cell receptor) [20]. CD56^bright^CD16^−^ and CD56^dim^CD16^+^ are two subsets of human conventional NK cells based on CD16 expression. The CD56bright NK cell population produces a large amount of IFN-γ and proliferates in response to cytokines including IL-2, IL-12, IL-15, and IL-18, but it does not respond to target cell recognition and has a low killing potential. CD56 deficient (CD56^−^) NK cells, on the other hand, are mostly cytotoxic and produce less IFN-γ [21,22]. A new fraction of NK cells has recently been discovered, i.e., a CD56^low^CD16^low^ NK cell subset with a surface receptor profile that is halfway between CD56^bright^ and CD56^dim^, as well as a strong ability to produce IFN-γ and cytotoxicity [23].

### 2.2. The Origin of uNK Cells

For a long time, researchers have been curious about the origin of uNK cells during pregnancy. To see if mouse uNK cells in the pregnant uterus grow in situ from progenitor cells in the virgin uterus or migrate there from the periphery, researchers performed uterine segment transplantation and adoptive transfers [24]. In a study, the uterine horn from a NK cell-competent mouse was orthopedically transplanted into a NK cell-deficient mouse and it was found that decidualized grafts included uNK cells only in the recipient mouse which suggests that the pre-uNK cells in the uterus do not self-renew [25]. Similarly, adoptive transfer of SCID mice’s bone marrow, thymus, lymph node, and spleen cells, as well as fetal liver cells, to lymphoid recipients resulted in the identification of donor-derived uNK cells in the pregnant uterus, demonstrating that NK or progenitor cell homing is conceivable [26]. According to a murine study, NK cell formation and maturation in the uterus share some characteristics with NK cell development in other tissues, but they also exhibit tissue-specific control as they help to shape the structural changes that occur during maternal-fetal interface development. It has also been suggested that immunological processes establish a balanced environment for uNK cell activation, proliferation, and differentiation through complex signaling pathways [27,28].

Previously it has been reported that there are few circulating CD49a^−^ DX5^+^cNK cells in murine virgin uteri but a large population of CD49a^+^ DX5^−^trNK cells [7]. These trNK cells are different from the other trNK cells found in the tissues of liver and skin as they lack the expression of T-bet transcription factor and identify as a distinct lineage of NK cells [24]. Furthermore, discovery of a high percentage of trNK cells in the virgin uterus raised the possibility that through local proliferation during pregnancy, they could contribute to the accumulation of uNK cells. In addition, parabiosis tests with experimentally induced decidualization in an early pregnancy model confirm that at the time of local proliferaton of trNK cells, migratory cNK cells contribute less [29].

In contrast, the data collected by Sojka et al., 2018 [30] don’t really ignore cNK cells’ contribution as they also accumulated in number from the periphery. As a result, the authors proposed a two wave hypothesis to support the contribution of both cNK cells and trNK cells to the pooling of uNK cells during pregnancy. The first wave begins during endometrium remodeling at the start of the decidualization process. This wave is caused by local trNK cell multiplication, with circulating NK (cNK) cells contributing minimally to the growing pool of uNK cells in early pregnancy, according to the findings [29]. The second wave involves the recruitment of cNK cells in placental processes, including vascular remodeling. Mice that lack cNK cells but retain trNK cells, such as Nfil3 mouse, show a significant defect in uNK cell accumulation and the placenta is suboptimal, with abnormal remodeling of the spiral artery [5,24,30,31]. Collectively these findings imply that trNK cells and peripheral cNK cells both play a role in the pooling of uNK cells during pregnancy.

## 3. Phenotype and Subsets of dNK Cells

### 3.1. Surface Receptors of dNK Cells

The dNK cells (uNK cells localized to the decidua basalis) are a separate population from the blood counterparts, according to the phenotypic characteristics and functional analysis during the first trimester in humans. The uNK cells exhibit a CD56^bright^CD16^−^ surface phenotype and share characteristics with both the subsets of NK cells (i.e., CD56^+^CD16^+^ and CD56^+^CD16^−^ NK cells) found in the peripheral blood. The uNK cells are less cytotoxic then the CD56^bright^CD16^−^ NK cells seen in the peripheral blood, as they do not kill trophoblast cells and are only weakly cytotoxic against cancer cell lines [32]. In early pregnancy, they make up 50–70% of all immune cells, which is higher than the number of NK cells in the blood, and they have a great capability to release a variety of cytokines and chemokines which in turn regulate numerous types of surrounding cells [33]. The dNK cells display tissue-resident markers such as CD9, CD69, and CD49a, as well as adhesion molecules including CD9, CD62L, and α-1 integrin, which may help them to accumulate [34,35]. They also contain higher levels of lysozymes such as perforin [36]. Furthermore, these cells express high levels of killer cell immunoglobulin like receptors (KIRs) and NKG2A, which can bind to different types of HLAs on trophoblasts, implying that dNK cells are involved in regulating trophoblast biological behavior [37]. Despite the fact that dNK cells express several activating receptors including NKp30, NKp44, NKp46, NKG2D, NKG2C, DNAM-I, and CD244, and have intact cytolytic machinery, they do not polarize the microtubule organizing centre and cytolytic granules in the area of contact with target cells, instead secreting a variety of soluble mediators spontaneously [38]. They express significant numbers of αEβ7, α1β1, αXβ2, and αDβ2 laminin receptors, but not the α6_β_1 laminin receptor. In addition, they produce large quantities of tetraspan 5, CD151, and CD9 tetraspanins, which in turn bind to integrins and influence the activity of integrin and its β5 subunit [39]. Furthermore, CCR1, CCR3, CXCR3, and CXCR2 chemokine receptors are expressed at higher levels during the first trimester in human dNK cells, while CCR7, CXCR4, and CX3CR1 chemokine receptors are expressed at lower level [39,40].

The uNK cell phenotype of non-pregnant endometrium is still not examined as thorough as that of pregnant endometrium, where phenotypic and functional variations across gestational ages have been documented. In a study of CD56^+^ cells obtained from non-pregnant uterus during various menstruation cycle phases, the expression of many cell surface markers was investigated, finding low to non-existent expression of HML-1, L-selectin, CD3, CD8, CD16, and CD25 (IL-2 receptor-α). However, from the proliferative to the late secretory phase of the menstruation cycle, CD2, CD49a, and CD122 expression rise, but CD11a, CD69, and CD49d expression remain constant, albeit CD49d level is significantly low in early pregnancy [41]. Furthermore, microarray studies comparing non-pregnant and pregnant endometrial CD56^+^ cells, discovered 450 genes that were differentially expressed with >twofold difference, with 70% over-expressed in the non-pregnant uNK cell subset, implying that non-pregnant uNK cells are far from inactive and are likely to play an important role during implantation and early placentation [42].

### 3.2. Subsets of dNK Cells

The dNK cells produce about 50–70% of immune cells at the maternal–fetal interface and play a number of roles during pregnancy. As a result, individual cells must have heterogeneity. Discovery of three primary pools of the dNK cells (dNK1, dNK2, and dNK3) with varied immunomodulatory features, as well as tissue-resident markers CD49a and CD9, which are expressed by all of these cells, bolstered these findings [43]. dNK1 pool expresses B4GALNT1, CD39, and CYP26A1 proteins. CD39 is a regulatory ecto-ATPase that helps to change the balance from a pro-inflammatory to an anti-inflammatory (immunosuppressive) environment. In addition, they have a higher expression of KIR2DL1, KIR2DL2, and KIR2DL3 (inhibitory receptors that can bind to HLA-Cs on trophoblasts) and KIR2DS1 and KIR2DS4 (activating receptors that can bind to HLA-Cs on trophoblasts). Furthermore, the expression of LILRB1, a high affinity receptor for the HLA-G dimer, and active glycolytic metabolism, suggest that the dNK1 subset interacts with fetal extravillous trophoblasts (EVTs) [8,44]. The expression of ANXA1 and integrin beta-2 (ITGB2) proteins define the dNK2 pool. The activating receptors NKG2C and NKG2E, as well as the inhibitory NKG2A receptors are found in both dNK1 and dNK2 cell subsets, suggesting a role of dNK1 cells in recognition and interaction with EVTs [42]. The dNK3 cells are characterized by the expression of CD160, CD161, KLRB1, TIGIT, CD103, and ITGB2 but the expression of innate lymphocyte cell markers CD127 is absent on them [8,44].

Based on the expression of MKI67 and TOP2A, another study classified dNK cells into resting and proliferating NK cells [45]. Proliferating NK cells are more involved in the cell cycle, organization of cellular components, and proliferation of cells than resting NK cells. On the other hand, the immunological response, cytolysis, and molecular function modulation are especially important for resting NK cells [45]. The protein CD49a (a tissue-specific marker) is expressed by the majority of dNK cells. The CD49a^+^Eomes^−^ Type I innate lymphocytes and CD49a^+^Eomes^+^trNK cells are two types of cells found in mouse dNK cells [8]. The CD49a^+^Eomes^+^trNK cells play important role in supporting placental and embryo development by secreting growth factors [46].

## 4. Non-Pregnant Female Reproductive Tract NK Cells

NK cells are important in the body’s innate immune responses. They can be found in the human spleen, lymph nodes, blood, lung, liver, gut, and endometrium. They show up as loose clusters in the stratum functionalize in the EM, adjacent to the upper endometrial glands and luminal epithelium [47]. They account for a considerable fraction of the leukocyte population in the human endometrium during the secretory phase of the menstrual cycle [47,48]. In human endometrium they emerge in a predictable pattern, and the amount of NK cells increases as the menstrual cycle proceeds [49]. These findings support a number of recent studies [47] that suggest sex hormones govern NK cell migration into the endometrium. During the pre-ovulatory, proliferative (follicular) phase of the menstrual cycle, a few small, agranular NK cells can be seen in the endometrium [50]. Their numbers increase significantly after ovulation under the influence of progesterone and they perish two days before menstruation when the level of progesterone decreases [51]. In the mid-late secretory (luteal) phase, they multiply quickly and become granular, where they come across with the spiral vessels and endometrial glands [52]. According to some researchers, when uNK cells die before menstruation, soluble factor products that maintain vascular integrity are reduced, which may lead to a monthly breakdown of endometrium which results in the menstrual cycle, implying that uNK cells may also play a role in endometrial renewal, differentiation, and disintegration [53,54].

It has also been shown that the cytotoxicity of endometrial NK cells is less than that of peripheral blood NK cells in the late proliferative phase [55]. In addition, proliferating uNK cells had greater levels of the activation markers CD69 and HLA-DR [56]. These characteristics of NK cells are hypothesized to have a function in preventing microbial infection. The cytotoxicity of NK cells, on the other hand, is reduced during the secretory phase, showing that local functional regulation is engaged at this time. They have also been shown to express mRNA for vascular endothelial growth factor-C (VEGF-C), placental growth factor (PGF), and angiopoietin-2 (Ang-2) during the secretory phase, all of which potentially influence the stability of arteries [57].

## 5. NK Cells in Pregnant Female Reproductive Tract

### 5.1. NK Cells during Key Early Events of Pregnancy

**Figure 2:**
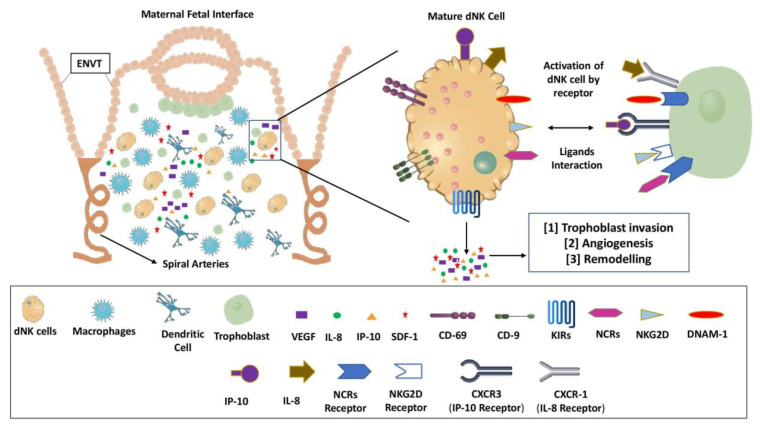
Interaction of trophoblast and decidual NK (dNK) cells at the maternal-fetal border. The interactions between the trophoblast and dNK cells are mediated by multiple interactions of receptor ligands at the interface between the mother and the fetus. This leads to activation of dNK cells, and the activated dNK cells release the cytokines and chemokines that play vital role placental development by mediating trophoblast invasion, angiogenesis and remodeling of placental tissues/spiral arteries. VEGF: Vascular endothelial growth factor; IL-8: Interleukin-8; IP-10: Interferon inducible protein-10; SDF-1: Stromal cell derived factor-1; CD-69: Cluster of Differentiation-69; CD-9: Cluster of Differentiaton-9; KIRs: Killer Ig-like receptors; NCRs: Natural cytotoxicity receptors; NKG2D: Natural killer group 2D; DNAM-1: DNAX accessory molecule.

The number of dNK cells increases during the mid-secretory phase and early pregnancy, until the end of the first trimester, and starts to decline as the fetus approaches term [58]. The dNK cells help the trophoblast migration by boosting blood flow at the fetal-maternal contact. Trophoblast cells have been discovered to express ligands for key NK receptor activators. These receptor/ligand interactions mediate the molecular contact between dNK cells and trophoblasts, and activated dNK cells release VEGF and stromal cell derived factor-1 (SDF-1) that are important in angiogenesis [36] (Figure 2). dNK cells secrete angiogenic factors such as VEGF and angiopoietin-2, as well as cytokines and growth factors such as TNF-γ, IL-10, GM-CSF, PlGF, IL-1, TGF-1, Colony stimulating factor-1 (CSF-1), leukemia inhibiting factor (LIF), and IFN-γ during pregnancy [59]. The VEGF, a potent growth factor implicated in angiogenesis and microvascular hyperpermeability, may have a role in creating localised angiogenesis during the establishment of the decidua/mesometrial lymphoid aggregation during pregnancy (MLAp). According to one study, human endometrium expresses mRNAs encoding VEGF-C, placental growth factor (PlGF), angiopoietins like angiopoietin 1 (Ang1) and Ang2, and the receptors VEGFR-3 (Flt-4), Tie 1, and Tie 2. Furthermore, the fact that VEGF-C, PIGF, and Ang2 expression was restricted to uNK cells only suggests that uNK cells may play a role in endometrium angiogenesis and regeneration [57]. The concentration of IFN-γ required at the implantation site for induction of spiral artery remodeling has been addressed by IFN-γ production by dNK cells during decidualization and normal pregnancy [60]. An in vivo study on transgenic mice lacking IFN- revealed that IFN-γ derived from dNK cells alters gene expression in the uterine vasculature and stroma, causing vessel instability and facilitating pregnancy-induced remodeling of decidual arteries [61]. However, it is unclear what role is played by IFN-γ, derived from dNK cells in humans and other nonhuman primates.

In purified human invasive trophoblasts, researchers discovered that dNK cells isolated from human decidua express IL-8 and IFN-inducible protein (IP-10), as well as the chemokine receptors for these ligands, CXCR1 (IL-8 receptor) and CXCR3 (IP-10 receptor) (Figure 2). In comparison to peripheral blood NK cells, the ability of dNK cells to stimulate trophoblast migration in an in vitro trophoblast migration experiment was greatly reduced in the presence of neutralizing antibodies IL-8 and IP-10. Overall, these studies revealed that the cytokines IL-8 and IP-10 generated by dNK cells can favourably limit trophoblast invasion [59]. Controlling EVT invasion into the maternal decidua, on the other hand, is crucial for placenta development and pregnancy outcome. Preeclampsia, fetal growth restriction (FGR), pre-term labour, and recurrent miscarriage can all be major side consequences of insufficient invasion [62]. Human dNK cells, on the other hand, have been shown to prevent trophoblasts from infiltrating the body. Human dNK cells isolated from early human pregnancy decidua generate IFN-γ, which limits trophoblast invasion by increasing extravillous trophoblast cell death and decreasing MMP-2 trophoblast synthesis [63]. As a result, in early human pregnancy, a fine balance is essential to avoid either under-invasion or over-invasion of trophoblasts.

Significant vascular remodeling is required to support placentation and fetal growth during early pregnancy in humans, with the decidual spiral arteries being replaced with larger-diameter vessels with low resistance and high flow that can provide nutrients and oxygen to the fetus [64]. Furthermore, placental EVT cells replace the endothelium of these veins, allowing blood flow to be diverted into the area around the placental villous tree and thus allowing nutrition and gas exchange between mother and fetus [65]. In both mouse in vivo and human in vitro experiments, dNK cells were found to play a significant role in vascular remodeling [66]. Many vascular abnormalities associated with implantation sites have been demonstrated, including thickening of the media and stroma, damage to the endothelium, reduced size of the placenta, and onset of stillbirth on day 10 of pregnancy [67]. Studies showed that transplantation of bone marrow from severely combined immunocompromised mice (lacking functional T and B lymphocytes) into NKcell-deficient mice restores the recipient’s uNK cell population, reduces vascular abnormalities, increases the size of the placenta, and restores fetal viability; this suggests that dNK cells play an important role in fetal development [68]. Overall, these findings support the idea that murine dNK cells play an important role in decidualization, placentation, and the proper vascularization of implantation sites.

### 5.2. An Overview of the Maternal-Fetal Interface Development

The maternal-fetal interface is the junction of the uterine mucosa and extraembryonic tissue of the developing conceptus [69]. It serves as a platform for the communication between two allogenic entities. The invasion of trophoblastic cells initiates the formation of the maternal-fetal barrier. These trophoblastic cells are derived from the blastocyst layer of the embryo as well as the endometrium. During human embryonic development trophoblast cells differentiate into two subsets: villous trophoblasts (VTs) and extravillous trophoblast cells (EVTs) [70]. EVTs invade blood vessel linings, and reconstruct the spiral artery, whereas VTs form chorionic villi, which are located over the surface of the villi and help in the transportation of nutrients and oxygen to the fetus. The expression of maternal and paternal genes, including major histocompatibility complex (MHC) MHC-like molecules, which allow mutual recognition by decidual NK cells, T lymphocytes, and dendritic cells, is required for trophoblastic cell invasion in the embryo and at the maternal interface [71]. Trophoblasts are the most common type of cells of the extra-embryonic tissues, and they help in the formation of placenta on one side of the conceptus and the chorioamniotic membrane on the other side. The lining of the uterus does not participant passively in the implantation of the embryo; rather, it goes through a special tissue reaction known as decidualization to aid the development and function of the embryo placenta [72,73].

Decidualization is a vital process in the developing maternal-fetal relationship. It is characterized by the proliferation and differentiation of mucous membrane stromal cells (ESCs) into massive spherical cells with extensive cytoplasm, as well as nuclear and phenotypic changes in the decidual stromal cells. With expansion of the embryo and invasion of the cytotrophoblast cells, decidua originating in the spiral artery spread across the endometrium [74,75]. The uterine glands, stromal cells, and immune cells all work together to achieve decidualization. The decidua’s hallmarks in early pregnancy include an increase in immune cells, which can account for upto 40% of total decidual cells, and the uterine glands’ histiotrophiccare of the growing placenta [75,76,77]. One of the most important requirements for optimal embryo development is endometrial decidualization. Infertility, recurrent or recurring spontaneous abortions (RSA), intra-uterine growth restriction (IUGR), and some other pregnancy disorders can be caused by a lack of decidualization [78].

### 5.3. Role of dNK Cells at Maternal-Fetal Interface

The dNK cells are widely available in the uterus and in close contact with trophoblasts. Although the exact functions of these cells are not known yet, according to some theories, they may contribute to implantation and placental development [79]. The presence of dNK cells at the maternal-fetal interface during implantation in many species, suggests that trophoblasts are target cells for dNK cells. dNK cells make upto 70% of lymphocytes during early stages of the pregnancy and are thought to have unique physiological activities. Chemokines, cytokines, and various angiogenic growth factors are secreted by dNK cells, which control the invasion of the trophoblasts and remodeling of spiral arteries [59,80]. According to a study, a number of soluble substances, notably GM-CSF, are secreted after co-culturing KIR2DS1^+^ dNK cells with HLA-C2^+^ target cells in vitro, which boosts the invasive capacity of the trophoblast cells [81]. In additional, dNK cells boost T_reg_ cells and the creation of indoleamine 2,3-dioxygenase (IDO, a vital negative regulator of immune responses) producing monocytes, induce effector T cell apoptosis, and contribute to the development of decidua basalis of mouse [61,82,83,84] (Table 1). These findings show that dNK cells operate as messengers during pregnancy by controlling local inflammatory reactions and endurance.

KIRs, CD94/NKG2A, and ILT2 expression in dNK cells is substantially higher than in peripheral blood NK cells (pbNK)during early pregnancy, and these receptors easily integrate with the HLA-C, HLA-E, and HLA-G molecules, respectively [85] (Table 1). To understand the involvement of NK cells within the maternal-fetal interface needs a close analysis of their reactions after KIRs create various individual combinations with HLA-C. NK cells are blocked when maternal dNK cells, such as the KIR AA haplotype, are joined with HLA-C1 from maternal self and HLA-C2 from paternal genetic feeding cells, raising the risk of preeclampsia. Because the mother has inhibitory KIR2DL1 receptors but not activated receptor KIR2DS, this is the case [86]. When the mother’s KIR2DL1 interacts with the embryo’s HLA-C2, dNK cells are suppressed, unable to produce cytokines, in direct contact with EVT, and do not adequately support EVT invasion into vascular arteries. The combination of the KIR-A haplotype gene KIR2DL1 and HLA-C2, which can cause placental defects, produces strong inhibitory signals. Thus, the capacity of KIR/HLA-C to work together will affect the ability of dNK cells to generate chemokines and cytokines, as well as the critical ability to regulate trophoblastic cell invasion [86,87]. Because KIR2DS2 is an active receptor that can be blocked by KIR2DL1, the KIR2DL2 and KIR2DS2 (both of which belong to the KIR-B haplotype) can reduce the frequency of KIR2DL1 expression and promote NK cell activation. Although both KIR2DL2 and KIR2DL1 are inhibitory receptors that can combine with HLA-C2, KIR2DL2 has a lesser binding force and inhibition effect. During the development phase of NK cells, KIR2DL2 expression takes precedence over KIR2DL1. Reduced KIR2DL1 expression levels also increase the ability to activate NK cells and reconstruct blood vessels, which may reduce the risk of preeclampsia. Even if the mother has KIR haploid 2DS1 and at the same time carries HLAC2 embryos, the embryo can be overweight and hinder labour [87,88]. In addition, HLA-E and NKG2A binding inhibit NK cell activation and keep trophoblasts alive; while binding of HLA-G and ILT2 promotes the secretion of inflammatory and angiogenic cytokines by decidual NK cells, such as interleukin-1 (IL-1), IL-6, IL-8, and tumour necrosis factor-α (TNF-α) [89]. Moreover, the previous research has revealed that appropriate dNK cell physiological performance ensures a healthy pregnancy, but decreasing dNK cell function results in pregnancy failure. Despite the fact that the actual etiology of dNK cell malfunction is unknown, maintaining the right activation status of NK cells is crucial for each normal pregnancy. Consequently, inhibiting NK cells function in therapeutic treatment may increase the risk [90].

**Table 1:** Crosstalk at maternal-fetal interface by combination of KIR and HLA from mother and fetus, respectively, and effects.

Maternal Phenotype	Fetal Phenotype	Effects	References
KIR2DS1^+^	HLA-C2^+^	Increase trophoblast cell invastion potential. Boost regulatory T cells. Create IDO producing monocytes. Induce effector T cell apoptosis	[71,81,82,83,84]
KIR AA	Homozygous HLA-C2 or Heterozygous HLA-C1C2	Increase the risk of preeclampsia	[86]
KIR2DL1	HLA-C2	Repress dNK cells and enable to produce cytokines Mediate strong inhibitory signals leads to the placental abnormalities. Affect the ability of dNK cells to regulate trophoblastic cell invasion.	[86,87]
NKG2A	HLA-E	Inhibit NK cell activation.	[89]
ILT2	HLA-G	Promotes the secretion inflammatory & angiogenic cytokines by dNK cells.	[89]

## 6. NK Cell Dysfunction in Pregnancy Pathology

The presence of more uNK cells in the endometrium of women suffering from infertility has raised the hypothesis that they are involved in the pathogenesis. However, the findings of various research studies are mixed, and despite the widespread use of cell count measurements in clinical research, currently there is no conclusive proof that uNK cells are involved in pregnancy pathology.

### 6.1. Preeclampsia (PE)

The pregnancy disorder PE is linked to trophoblast invasion and unsuccessful spiral artery transformation [91]. As the dNK cells are connected to trophoblast invasion control and promotion, as well as the early stages of spiral artery transformation, it is plausible that changes in dNK cell numbers or function are involved in the etiology of these aberrant pregnancies. There is a link observed between PE, the paternal HLA-C molecule, and dNK cells, according to current studies. The HLA-C2 allotype in the fetus, as well as the lack of activating KIRs on maternal dNK cells, have both been demonstrated to increase the risk of PE. Poor trophoblast invasion and spiral artery remodeling, both of which are hallmarks of PE, were assumed to be caused by the absence of this activating KIR [92,93]. Some studies have found that women with PE had more CD56^+^ dNK cells in their decidua than age-matched controls [94,95,96]. The PE on the other hand, was associated with a reduction in CD56^+^ dNK cells, according to an immunohistochemical examination of placental bed biopsies [97]. These studies, however, are restricted in their utility because they measured dNK cell numbers after birth, and it is uncertain whether the results reflect cell levels earlier in the pregnancy. It is most intriguing that paternal HLA-C/maternal KIR interactions play a crucial role in the pathogenesis of PE. A small number of dNK cells from high-risk pregnancies express KIR2DL/S1, 3 and 5, and LILRB1 too, suggesting a different interaction with extravillous trophoblast via class I MHC antigens [98].

In a recent genome-wide association study of 7219 PE cases, it was reported that the maternal HLA-B allele, which fails to educate NKG2A^+^ NK cells, is associated with a 7% higher relative risk. The authors also suggest that inactivation/deletion of NKG2A in mice resulted in aberrant angiogenesis during pregnancy (a key feature of PE) [99]. A study by Caniggia et al. has reported that TGF_β_-3 expression in the placenta is high during the early phase of pregnancy and drops by the 9th week of gestation. The over expression of TGF_β_-3 has also been demonstrated in preeclamptic placenta, suggesting that this failure in the down regulation of TGF_β_-3 expression may lead to the preeclamptic pregnancy [100]. Recent flowcytometric analysis of decidual samples obtained from three different groups of women has concluded that increased TGF-_β_ expression in decidua down regulates the dNK cells activation, leading to PE [101]. In addition, a number of studies have also linked PE to changes in the number of dNK cells. Some studies have found that the number of dNK cells is much larger in PE pregnancies than in normal pregnancies [96,102], whereas others have found the reverse [103]. As no conclusion has been derived due to heterogeneity in the results from different studies, further research is required.

### 6.2. Recurrent Implantation Failure (RIF)

When there is evidence that implantation has not occurred, it is usually diagnosed as implantation failure. “Three or more unsuccessful treatment cycles” is the most often used term, followed by “two or more failed treatment cycles” [104]. RIF is frequently caused by faulty endometrium, and functional investigations have revealed that uNK cells play an important role in the projection of implantation period, suggesting that they may contribute to the failure of an embryo implantation [105]. Reports also reveal that increased numbers of uNK cells are present in pre-pregnancy endometrium in conjunction with RIF when compared to normal fertile controls in a flow cytometry analysis of young individuals with a history of repeated unexplained implantation failure [106].In addition, in the mid-secretory phase, women with RIF after IVF show a high increase in uNK cells as a proportion of stromal cells, with uNK cell numbers correlating to stromal expression of IL-15, compared to normal fertile controls [107,108]. Similarly, a cohort study found that women with RIF possess higher endometrial IL-12 or IL-18 levels which might be linked to higher uNK cell numbers. This group also showed aberrant uterine arteries doppler compared to the controls or RIF women without increased uNK cells and cytokines [109].

Recently, a study found no significant differences in the quantity and distribution of uNK cells relative to endometrial arterioles in women with RIF compared to women who had successful embryo implantation after IVF [110]. RIF risk is also linked to haplotypic polymorphisms in KIR genes, just like preeclampsia. Women with the maternal KIR AA haplotype were more likely to experience RIF after IVF therapy than those with the KIR AB or KIR BB haplotypes [111]. In a systematic analysis, comparing women with RIF to controls revealed no difference in pNK or uNK cells in the endometrium. The heterogeneity of the studies once again prevented any meaningful meta-analysis of the findings [112]. As a result, testing for pNK or uNK cells in the setting of RIF needs further investigation.

### 6.3. Recurrent Miscarriage (RM)

The pregnancy disorder RM is defined as “the loss of three or more consecutive pregnancies (determined by a positive pregnancy test) before 24 weeks of gestation” [113] or “the loss of two or more pregnancies in a row before 24 weeks of gestation” (identified by a positive pregnancy test) [114]. RM has a wide range of causes which can be divided into maternal and fetal categories. Maternal causes include pre-existing medical conditions such as antiphospholipid syndrome and polycystic ovarian syndrome, as well as uterine abnormalities and fibroids. Chromosome abnormalities, which can be hereditary or develop spontaneously, are one of the most common fetal causes [115].

Many immunohistochemical studies from various groups have found that women with a history of RM have a higher number of uNK cells in the mid-secretory phase endometrium [116,117,118]. In 1999, Quenby and colleagues spotted significantly higher number of uNK cell in women with subsequent miscarriage as compared to those who had a live birth, [112] but another study failed to find such a connection [108]. A few studies have not found any differences in endometrial uNK cell counts in women with RM [119,120,121]. Despite some variations in results, immunohistochemical examinations have consistently demonstrated greater uNK cell numbers in mid-secretory phase endometrium of women with a well-defined history of RM. In addition, Chao et al. (1995) have reported increased activity of the uNK cells in decidua associated with RM [122]. There are also mixed results from investigations on uNK cells in decidual tissue from women with RM who have lost several pregnancies. The number of NK cells increased in one study, whereas their number decreased in another study [123,124]. In each of these investigations, tissue following a miscarriage was compared to tissue from women who had their pregnancies terminated voluntarily; changes might thus be a result of the miscarriage rather than a cause.

In a study comparing dNK cells from age-matched healthy controls and RM patients, a lower level of CD49a and higher expression of perforin, granzyme B, and IFN-γ has been spotted in RM patients. This study revealed that CD49a acts as a regulator of dNK cell functions like cytotoxic activity, migration, and adhesion. Moreover, the long non-coding RNA lnc-49a acts as a positive regulator of CD49a in human dNK cells [125]. A study by Guo et al. revealed that individuals with RM had decreased KIR2DL4 expression on dNK cells and lowered HLA-G expression on trophoblasts, resulting in dNK cell pro-invasion and pro-angiogenesis activities being impaired [126]. The mixed results from these trials show that uNK cells might play a crucial role in pathogenesis of RM but further investigations are required to confirm the findings.

## 7. Immunomodulatory Strategies for Treatment of Pregnancy Complications

During a normal pregnancy, the maternal immune system and fetal antigen work together, and failure of this immune system adaptation lead to alloimmune rejection of the fetus, resulting in various pregnancy-related complications. In order to positively modify the maternal immune system or decrease an excessively damaging immune response, immunomodulatory medicines such as glucocorticoids, intravenous immunoglobulins, and anti-TNFα medications are used in the treatment of pregnancy related complications such as RM [127]. In a study, prednisolone was tested on women with idiopathic RM, and was noted that to reduce the number of uNK cells in preimplantation endometrium, which is found to be one of the reasons leading to the RM [128]. Moreover, a study showed that significantly higher number of pNK cells were present in idiopathic RM patients as compared to the controls [129]. This study concluded that pre-pregnancy immunomodulatory treatment in RM patients may be beneficial in lowering pNK levels and establishing a supportive immunological environment for fetal development [129]. Furthermore, a study analyzing the immunomodulatory effects of menstrual blood-derived stromal/stem cells (MenSCs) on NK cells, revealed that MenSCs have inhibitory effect on NK cell cytotoxicity. The MenSCs were used as a surrogate for endometrial mesenchymal stromal/stem cells (eMSCs) in this study and it was implied that dysfunctioning of eMSCs may cause NK cell deregulation, leading to pregnancy related disorders like RM [130]. Recently, a review has described the use of curcumin as a therapeutic agent to manage pregnancy-related disorders due to its immunomodulatory effects [131].

## 8. Conclusions

Several researches have been undertaken on NK cells since it was revealed that they represent a critical leucocyte component of the endometrial stroma during implantation and early pregnancy. Although there have been significant advancements in understanding the role of NK cells, the availability of human tissues and the limits of extrapolating from animal models have limited investigations of very early stages of pregnancy. Functional studies of dNK cells have revealed their functions, with a particular emphasis on cytokine and angiogenic growth factor release, and spiral artery remodeling in early pregnancy. The researches have indicated that NK cell counts are altered in recurrent reproductive failure, but the functional implications are unknown. Before effective diagnostic and therapeutic approaches can be developed, it is critical to understand the mechanisms underlying the increased number of dNK cells, as well as the functional consequences. Furthermore, the possible contribution of male variables such as seminal fluid components to uNK cell differentiation and function in recurrent reproductive failure should be investigated in future studies. In addition, knowledge of cross-talk between diverse cell types in the uterine milieu is limited, and future research should also focus on elucidating the complexity of these interactions in various physiological and pathological circumstances.

## Data Availability

Not applicable.
